# THE MAKING OF AFRICAN MADE: MEMORY AND DEMENTIA EDUCATION BY AND FOR THE AFRICAN IMMIGRANT COMMUNITY

**DOI:** 10.1093/geroni/igad104.1021

**Published:** 2023-12-21

**Authors:** Manka Nkimbeng, Truphosa Aswani, Wynfred Russel, Tetyana Shippee, Joseph Gaugler

**Affiliations:** University of Minnesota, Minneapolis, Minnesota, United States; African Career Education And Resources Inc. (ACER), Brooklyn Park, Minnesota, United States; African Career Education And Resources Inc. (ACER), Brooklyn Park, Minnesota, United States; University of Minnesota, Minneapolis, Minnesota, United States; University of Minnesota, Minneapolis, Minnesota, United States

## Abstract

African immigrants are a fast-growing segment of the U.S. Black population but dementia literacy and care needs of this population are not fully understood. A dementia care assessment in the community highlighted dementia education as a high priority need in the African immigrant community in Minnesota. This presentation will describe the collaborative process between the research team and a community project advisory board (PAB) to develop a culturally tailored dementia education program and booklet. Following several meetings and reviews, the PAB approved an interview guide that was used to conduct 10 key informant interviews (with leaders including priest, executive directors etc.) and three community conversations (30 participants). This data was used to identify important cultural dimensions for incorporation into the education program. Themes identified were need for respect, attitudes, beliefs and challenges related to dementia care. Identification and debunking of dementia and dementia care myths were also highlighted. The resulting educational content has been presented at 3 community events/organizations with many more scheduled in spring 2023. The African community in Minnesota is very receptive of the education program with many individuals noting that it truly captures African immigrant cultural norms and values. The collaborative process between the research team and the advisory board could serve as a model for creating culturally tailored dementia education programs in other communities. Additionally, successful implementation of this education program has implications for improving dementia care among African immigrants and reducing health disparities in this population.

